# Digital Identity Infrastructures: a Critical Approach of Self-Sovereign Identity

**DOI:** 10.1007/s44206-023-00049-z

**Published:** 2023-05-11

**Authors:** Alexandra Giannopoulou

**Affiliations:** University of Amsterdam, Institute for information law (IViR), Amsterdam, the Netherlands

**Keywords:** Digital identity, Self-sovereign identity, Trust, Blockchain, Control

## Abstract

The shift from electronic identification to digital identity is indicative of a broader evolution towards datafication of identity at large. As digital identity emerges from the fringes of technical challenges towards the legal and socio-technical, pre-existing ideologies on the reform of digital identity re-emerge with a newfound enthusiasm. Self-sovereign identity is one representative example of this trend. This paper sets out to uncover the principles, technological design ideas, and underlying guiding ideologies that are attached to self-sovereign identity infrastructures, carrying the promise of user-centricity, self-sovereignty, and individual empowerment. Considering the flourishing of digital identity markets, and the subsequent institutional interest on a European level in the techno-social promises that this identity architecture carries, this paper explores how the implementation of EU-wide self-sovereign identity shifts the already existing historical power balances in the construction of identity infrastructures. In this contribution, we argue that the European-wide adoption of self-sovereign ideals in identity construction does not address the shortcomings that identity and identification have historically faced and that instead of citizen empowerment, it puts individuals (a category broader than citizens) in a rather vulnerabilized position.

## Introduction

When Cambridge Analytica’s data-extractive business practices were revealed in 2018,^1^ the online identities of more than 80 million Facebook users had already been compromised (Brescia, 2021). More recently, the newly installed Taliban government in Afghanistan has reportedly taken control of the digital identity infrastructure e-Tazkira,^2^ a biometric identity card used by Afghanistan’s National Statistics and Information Authority, which includes fingerprints, iris scans, and a photograph, as well as voter registration databases. What these two examples share is that they exemplify two distinct digital identity infrastructures, which concern different facets of what we consider identity, and that they highlight some of the complexities arising from its ensuing -inevitable- digitization.^3^ These examples are representative of security risks that can occur at a scale and speed previously unattainable (Beduschi, 2021), especially when digital identities escape the context for which they were created.

The acceleration in the design of digital identity solutions solidifies the need for creating trustworthy tools that are embedded in corresponding identity infrastructures. Control over digital identity and its ensuing infrastructures is key. We define digital identity infrastructures,^4^ as systems that construct, control, and commodify (facets of) user identities. These infrastructures (I) are formed by state actors and by private commercial actors, operating as identity providers; (II) construct identifiers with or without the direct control or intervention of the referred user; and (III) mediate these identities through technological design choices that are guided by identity providers who exercise power and control over them.

Digital identity can be generally understood as the representation of our identities in a machine-readable, datafied format. This process does not correspond to a single digital artifact, with a unitary function. As highlighted by Nyst et al., (2016, pp. 8–9), digital identity corresponds to systems of identification of individuals, as well as to systems of authentication that modulate access rights and authorize the performance of pre-specified actions or predetermined access to services. According to the authors, “the three functions of identification, authentication and authorisation are all performed digitally” (Nyst et al., 2016). This means that there is no offline process that corresponds to and facilitates any of the three aforementioned functions. This paper uses the above understanding of digital identity when discussing how it is implemented through self-sovereign technological infrastructures and corresponding ideologies. In doing so, we choose to leave outside the scope of this paper several understandings of digital identity, such as the derived/constructed digital identity or otherwise conceptualized as “corporatised identities” (Smith, 2020).

But what is self-sovereign identity? We have defined it as an “identity management system created to operate independently of third-party public or private actors, based on decentralised technological architectures, and designed to prioritise user security, privacy, individual autonomy and self-empowerment” (Giannopoulou & Wang, 2021). Although there is no consensus on the formal definition of this concept, authors agree that “self-sovereign” identity “aims to preserve the right to selective disclosure of different aspects and components of one's identity, in areas and different contexts” and that it refers to the idea that “individuals must retain control over their personal data and, to a certain extent, over the representations of their identities (or personas) within a particular identity management system” (Wang & de Filippi, 2020, p.9). It is therefore a question of giving the possibility to the person to determine and control who can access what information concerning them.

In legal terms, self-sovereign identity is often associated to the principle of informational self-determination.^5^ This principle—construed by the German Federal Constitutional Court in the 1983 Population Census Case—has been described as a precondition for a free and democratic society.^6^ However, while the first cumulatively understands and refers to identification as a techno-legal concept (Allen, 2016), the second is confined to the legal sphere as it is attached to fundamental rights of privacy and data protection. Capturing digital identity as an information transaction, the implementation of self-sovereign identity involves employing appropriate technological tools that attempt to maintain privacy, data protection, and security of the identification or information transfer process as these concepts are understood by the self-sovereign identity enthusiasts. All of the principles attached to this identity system, whether we think of confidentiality, integrity, availability of data, respect individual empowerment, and control, quickly became affiliated to blockchain-based systems (Giannopoulou, 2021; Gstrein & Kochenov, 2020).

Despite the relatively recent popularization of the technology, blockchain has been adopted in the relevant identity discourse as the appropriate technological ground based on which various self-sovereign identity systems can develop. In short, the expansion of self-sovereign identities is considered fundamental for blockchain enthusiasts because it could become the first successful implementation of blockchain-based systems following that of cryptocurrencies.^7^ Blockchains were originally developed as the necessary infrastructure to decentralize money and underlined the materialization of bitcoin. In these technical architectures, there is a clear link between the money and identity as evidenced by David Birch, who qualifies identity as the new money.^8^ This association led to the technology quickly capturing the interest of technical identity groups, who began to explore its potential application in ensuring disintermediated, secure, and decentralized digital identities.

Blockchains are designed to track and trace digital assets and their respective transactions through immutable ledgers. Their implementation in self-sovereign identity schemes aims to transpose these features by treating digital (self-sovereign) identity as a set of identification credential transactions that can be described as an architectural problem. Overall, and without attempting to go in detail over all key characteristics of blockchains, it can be said that an innovation element of this technology is the deployment of consensus algorithms that create security in decentralised peer-to-peer architectures. So, blockchains present the following principle-based characteristics: “(i) decentralised consensus, i.e., no central entity or third party is responsible for decision-making; (ii) immutable archive, i.e., an ordered list of transactions that cannot be removed or altered; (iii) transparency and verifiability, i.e., all recorded entries can be accessed and verified locally; (iv) resilience to failure” (Valiente & Tschorsch, 2021).

This paper will first succinctly provide an overview of self-sovereign identity, and it will describe the socio-technical apparatus that is created following key ideas and principles that are set to determine self-sovereign identity systems. The paper then takes a step back in order to position this development in broader theoretical and historical identity understandings that relate to its ensuing digitalization. Finally, the contribution follows the shift from state-wide digitalization of identity towards a European-wide network of identity infrastructures through the implementation of regulatory, policy, and technological tools. In this shift, self-sovereign identity becomes the stelar techno-social solution to the shortcomings of existing digital identity solutions. However, this way, as the paper contends, the already booming multi-billion-dollar digital identity industry^9^ appears to be able to drive and determine the development of the (public) digital identity infrastructures of the future.

## Understanding Self-Sovereign Identity

Providing an unanimously accepted definition of self-sovereign identity is far from a *fait accompli*. There are flagrant ambiguities in the socio-technical connotations of this concept, as evidenced by various attempts to break down its guiding principles into technological architecture guidance (Preukschat & Reed, 2021). From a historical point of view, this concept originated online among tech communities who came together around the topics of encryption and security, and who viewed the lack of a permanent, secure, and trusted layer of identification on the Internet as a problem to be solved with a technological solution. There are many promises attached to the evolution of identification online towards self-sovereign identity, and the potential it presents: “the SSI paradigm shift is also deeper than just a technology shift—it is a shift in the underlying infrastructure and power dynamics of the Internet itself” (Preukschat & Reed, 2021). The expectation is that this is more than a new technological implementation; it is a new technological revolution that will readjust existing powers and equalize them to the benefit of all (self-sovereign) individuals.

Viewed as a network connecting different machines on a planetary scale, the original design of the Internet did not leave any room for permanent digital identification of people in its technological design architecture. As the provision of online services proliferated, the creation of a trusted or even permanent digital identification presented a particularly interesting challenge, especially as it quickly became apparent that this identification infrastructure would have to incorporate particularities linked to different forms of individual identity. In practice, each individual has to create and maintain various different identities in the form of digital profiles (e.g., social identities, social security, educational identity, financial identity). Consequently, many problems related to the management of digital identification quickly appeared. Self-sovereign identity was created as a response or an alternative reality to these problems.

From a technological perspective, self-sovereign identity constitutes a technological architecture built to by design avoid the risks inherent in the current model of digital identification (Hoepman, 2021). This architecture is based on informal basic and abstract principles, which were identified by various technical communities exchanging on their frustrations and their aspirations relative to the identification of the future.^10^ These communities define self-sovereign identity as a set of ethical principles and an idealistic vision according to which individuals are “masters of their own identity” (Wang & de Filippi, 2020). The principles in question were systematized by Christopher Allen, whose aim was to establish a theoretical framework on the basis of which several self-sovereign digital identity systems could be put in place. The ten fundamental principles follow Kim Cameron’s laws of identity, namely, (1) existence, (2) control, (3) access, (4) transparency, (5) persistence, (6) portability, (7) interoperability, (8) consent, (9) minimization, and (10) protection. This list serves as a by design guide to self-sovereign identity. The principles are only completed by brief explanations, making any effort to concretize a specific self-sovereign identity system almost impossible. We cannot ignore the lack of consensus or certainty around what distinguishes a self-sovereign identity from an identity that is not self-sovereign.

The technical dimension of self-sovereign identity has so far been associated with decentralized identifiers (DIDs), verifiable credentials, and other related World Wide Web Consortium (W3C) standards, namely, the same standards body behind common Internet protocols like HTML and HTTPS. These identity decentralization standards constitute a set of technical standards which determine the methods of association of the data concerning an identified person in a persistent and universal way, so that this person not only has control over the way the information is linked and used, but also above all remains the master of its profile instead of a third-party service provider. Thus, all linked data can become globally portable, available to each individual in the form of digital certificates stored in a personal digital wallet. These certificates contain several types of information that identify an individual. Often, they grant access rights or privileges to the identified person. They can also be used for information verification, such as a link to identity documents, professional certifications, or any other data or information. If these technological elements related to the creation of digital identities exist independently of self-sovereign identity, it is the rise of blockchain that has, it seems, succeeded in creating a revival for the latter. This seems to be gradually imposing itself even though the advisability of using it deserves to be questioned in view of the risks it poses to data protection.

Finally, the fundamental characteristic of self-sovereign identity is the idea that it can be “the identity of a person which does not depend on nor is subject to any other power or state” (Preukschat & Reed, 2021, p.11). This aspiration aims to decouple the individual from external actor identity verification dependence. There are many paradoxes in this adage, all of which are representative of the conflation of identity with the technological forms it can embody over time. As we will clarify in the following sections, identity is expressed as a relationship between the individual and the collective, one which expresses various power dynamics between identifier and identified. Against this backdrop, self-sovereign identity is appearing as a simple identity technological artifact,^11^ a digital solution aspiring both to formalize the individualization of access to computer networks and to digitally recreate the relationships that (in)form individual identity.

## (Digital) Identity in Context

Our identities mark our belonging. We are because of our in corporis markers, such as our biometrics and DNA, and because of our own lived experiences of belonging. We exist in layered overlapping communities and are respectively perceived as members because of certain attributes operating as inscriptions and traces of our existence. Bauman recounts identity as “an idea” which aimed to “bridge the gap between the ‘ought’ and the ‘is’ and to lift reality to the standards set by the idea — to remake the reality in the likeness of the idea” (2004, p.20). For anthropologists, identity is expressed as a relation between the individual and the collective/population (M’charek, 2000). In this way, the individual comes to be clustered as part of, e.g., a gendered collective, a minority, and a vulnerable population.

Identity has frequently been used to highlight different facets of human self-definition (Gecas & Burke, 1995). However, it is not a stable pre-defined or rigid concept. According to Bauman (2004:15), identity “is revealed to us only as something to be invented rather than discovered; as a target of an effort, ‘an objective’”. Identity is an inscription from which leads a trail that can open up different paths. It has many facets, each one of which is formed, maintained, used, and exchanged based on different narratives. One individual can have several sets of attributes depending on the entity that is accumulating, inferring, or creating these attributes. Thus, “identity is not a given thing in the world, but it is a result of a process of construction, whether by the actor themselves or by others” (Khatchatourov, 2019, p.36).

Identity is foundational for societal mutual self-knowledge, since it “plays a central role in the enterprise of collective meaning-making, the realization of self-determination, the creation of social capital and societal trust” (Brescia, 2021). In both the physical and digital realms, we construct our identity through the selective self-disclosure of our traces and markers. This “process of making the self, known to others” (Jourard & Lasakow, 1958, p.91), i.e., self-disclosure, is the telling of the previously unknown so that it becomes shared knowledge (Joinson & Paine, 2009, p.2). The scope of this self-disclosure usually depends on the context in which it occurs, serving as a foundation of trust between individuals and the respective actors or between individual members of a group.

Selective self-disclosure does not imply that the revealed facets are “personae that some central self dons in its inauthentic mode. Rather these selves constitute the person. A person is something like a corporation of context-dependent characters” (Schoeman, 1984, p.409). The digital revelation of these different identities is assessed by both actors in the process based on the context, the necessary minimum level of trust, and the individual control over the revealed information. The disclosure implications in this self-narrative depend on the context of the revelation and the environment within which this occurs, which is why control over the separation of selves is fundamental. The power to keep our different identities (in their datafied form or other) distinct from each other is an important component of informational self-determination.^12^

We distinguish identification, i.e., the process of constructing, inscribing, and documenting identity to identity itself. These two concepts are interdependent, in that identification is scarcely thinkable without the use of categories of identity (Torpey, 2018) and categorization itself has been driven by the development of identification apparatuses.^13^ In the digital context, new technological architectures emerge and promise to deliver “efficient”, “secure”, “convenient”, and “user-centric” digital identities. Among these proposals, the self-sovereign identity technological proposal is claiming its space in the global identity market and identity policy-making.

These promises are centered on an individual empowerment narrative, one that often appears to use identity and identification interchangeably and that disregards the perpetual motion of our oft distinct identity facets and of our identification apparatuses. This conflation is not exclusive to self-sovereign identity, and it has far from faded with the development of digital identity infrastructures.

Lately, these identity infrastructures are becoming the locus of competition between commercial identity providers and institutional (public) ones. The empowerment narratives that permeate modern (identity, but also general-purpose) technological infrastructures—grounded on neoliberal ideals—are emerging in the EU policy discourse which is progressively populating these foundational infrastructures with liberal technological architectures attempting to empower technology users as citizens.

The different socio-technical understandings of identity influence the development of policy objectives on a European level. Witnessing the information inflation permitting the identification of an individual, the legislator was prompted to update identity-related regulatory frameworks to both improve efficiency and to protect citizens. Importantly, and as will be explained at a later section, digital identity is the subject of the proposal for a European regulation amending regulation (EU) No 910/2014 with regard to the establishment of a European framework relating to a digital identity.^14^ The objective is for a person to be able to electronically and securely transmit information concerning them throughout the European Union. The means of electronic identification referred to in the proposal, i.e., a national electronic identity card and a European digital identity wallet, are all based and rely on the legal identity of citizens. The European wallet goes one step further by allowing an individual to prove attributes such as holding a driver’s license or a diploma and to affix electronic signatures. It must also be able to be used to identify oneself to various players, in particular very large online platforms, so as to circumvent the means of identification developed by the latter, such as the “register/identify with Google” or “register/identify with Facebook” options. In doing so, the policy objective is to move from centralized or federated models to a user-centric model, with the person being placed at the heart of the decision-making process. It would then, according to the regulation in question, become self-sovereign.

Using “philosophically loaded phenomena” (Ishmaev, 2021) such as the above to describe an understanding of digital identity as technical identification and access control can easily lead to misconceptions because these can be formulated employing separate-yet-interdependent meanings. On a policy level, digital identity has been defined as “a collection of electronically captured and stored identity attributes that uniquely describe a person within a given context and are used for electronic transactions” (World Bank Group, GSMA and Secure Identity Alliance, 2016). This means that digital identity is often reduced to a “set of claims made by one [digital] subject about itself or another subject” (Cameron, 2005) or “the unique representation of a subject engaged in an online transaction” (Grassi et al., 2020). This risk of semantic misunderstanding is prevalent in self-sovereign identity systems, as its proponents attempt to clarify (by simplification) that the use of the terms is intended to only refer to a technological design (Wagner et al., 2018). Khatchatourov describes the double essence of the concept: “Digital identity can therefore have two complementary meanings, which precisely constitute the crux of the problematic of this domain: identification of the user and their actions in the digital environment and the effects of digital technology on the construction of identity understood as a relationship to oneself, to others and the public space” (2019, p.24).

This conceptual versatility—referring to any informational structure that represents any expression of the (or of a) self—is not new especially vis-a-vis theoretical approaches on personal identity and the self, the answers to which philosophy has been addressing throughout its history (Floridi, 2011). To bundle all different types of identity informational (infra)structures together would imply an admission that these can be considered interchangeable or that they can be regulated similarly. The conflation becomes particularly flagrant when one considers public infrastructures for digital identity provision. This would include public sector identification and the risks and challenges of which are rather higher than the ones from social identity infrastructures. In the following section in particular, we will showcase how the technological history of digital identity creation has shifted these risks and challenges from qualifying the necessary safeguards for trust-producing actors (Bodó, 2021) to ensuring the appropriate trust-mediating technologies.

## Shaping Public Digital Identity Infrastructures

Digitization has created a new class of external actors and parties who have the power of constructing and maintaining identities through their systems and technologies of categorization and discrimination. Online platforms, services, and digital technologies, which have the capacity to authenticate its users, can also use this function to collect, analyze the digital traces their users leave on their services and use that to build categories, and assign identities to those users. The “construction of personal identities in the infosphere” (Floridi, 2011, p. 550) is certainly not operating in a vacuum, but in a continuous interaction with the offline identity infrastructures and their corresponding power relations. The differences between the two become particularly relevant when one considers the integration of these power relations in the medium that constitute the digital identity itself. The choice, design, architecture, and governance of the technological artifacts building digital identity are rarely distinguishable from the identity itself. A brief look at the technological evolution of identities and their integration as socio-technical tools is necessary to illustrate the formation of the infrastructures, especially when these are stemming from state actors.

In technical terms, digital identity is split across “authentication” (who are you?) and “authorization” (what can you do?). As previously highlighted, it has been used interchangeably both with technologies of identification and identification management. While the first refers broadly to the practices and technological artifacts used to identify the person, the second describes all technical and organizational processes that ensure that only authorized and authenticated users can get access to the offered services. This conflation of meaning has preoccupied the role, responsibilities, and accountabilities of public institutions and the State, which have systematically been in charge of large-scale data accumulation and which are, by social consensus, established identity providers. The power aggregation that goes hand in hand with State-sponsored digital identity processes has not gone unnoticed. In France, since the first attempts to systematize digital identity for e-administration purposes, the concern over ensuring the accountability of the government materialized through the creation of the French Data Protection Authority (CNIL). This authority was created as an independent control and counter-weight mechanism when the French government decided to establish a centralized database that would uniformly manage e-administration processes. It was the public outcry and concerns over government overreach, surveillance, and the respect of fundamental rights that eventually led the government to create this independent authority through the law of 6 January 1978.^15^ The creation of the CNIL served as the auspice for the current model of data protection authorities, present in all member states.

Seen as technologies of identification, and thus as a combination of authentication and authorization, digital identities were popularized well before the advent of the World Wide Web. The evolution of telecommunication networks is marked by a foundational shift towards what would become an essential precondition to access networked services.

Historically, telephone use underwent a transition from operator service to rotary dialing, with all telephone owners being assigned a telephone user number that would go on to become one of the foundational and most consistent technological digital identities (Holt & Palm, 2021).^16^ The Internet exists as a vector of (technical) communication and connection between addresses referring to identifiable machines (computers) on a global scale. This technical capacity evolved to refer to individuals sitting behind the identifiable machine (Palfrey & Gasser, 2007) as personal computers became ubiquitous. A remarkable illustration of this shift is the normative discussion that led to the qualification of dynamic IP addresses as personal data.^17^ With online services proliferating, so did user accounts. This led to the need for online identity management, or the creation of account-based mechanisms that regulate access to online services and computer systems (Hoepman, 2021).

Eventually, identity provision became a service offered by big platform players like Google and Facebook. These companies benefited from the open design technical standards such as OpenID^18^ and OAuth^19^ (Maliki & Seigneur, 2014) to position themselves as identity providers for third-party web services and platforms. This social login—managing authentication and authorization on a horizontal level—rapidly became prevalent despite privacy concerns (Tene, 2013) and the loss of user control over the circulation of their data.^20^ The practical expansion of authentication systems to a growing number of service provision, combined with technological advances that permit and enable a better understanding of online user behaviour, led to the shift from systems of authentication towards systems of identity construction. As succinctly put by Denouël, “the development of the Internet has been accompanied by the emergence of devices more specifically dedicated to the production of the self and whose ordinary uses have provided fertile ground for the study of what is commonly called digital identity” (2011, p.75). David Chaum, the first cryptographer to explore applying cryptographic features to cash, argued that “computerization is robbing individuals of the ability to monitor and control the ways information about them is used” (Chaum, 1985). At the time, the necessary space for anonymity^21^ and pseudonymity (Bernal, 2014) was preserved,^22^ especially because the online presence of the State, i.e., government-mandated identification, had scarcely been developed—if at all.^23^

Digital state presence has significantly increased since the early internet years. The creation of a government identity layer is becoming a technical necessity for the continuous enjoyment of e-administration services especially in the current environment where government services appear to be proliferating and/or becoming available exclusively online. Substantially, this technical feature of digital identity is necessary as a risk-mitigation artifact because a trustworthy and secure digital identity means it is less prone to be appropriated, misused, and fraudulently presented.

As web-based services continued to grow, a new form of “state modernization” (Scott, 1999) started to gain momentum through digitalization (Hansen et al., 2018) and datafication (Mejias & Couldry, 2019) processes. The shift toward digital state service provisioning, with remote and secure citizen identification, was prioritized. This was due to many factors, including (1) a policy push towards the development of e-government services (Dagiral & Singh, 2020) and the growth of “platformised” state, where “infrastructures slowly turn into the « invisible background» of state-citizen interaction infrastructures”17 (Singh, 2019); (2) the penetration of social platforms, which brought about regulatory interventions such as content moderation rules significantly narrowing the available margins for anonymity and pseudonymity online; and (3) surveillance (Gürses et al., 2016; Beydoun, 2022).

The process of identification is based on the establishment of a classification system, a categorization of individuals that determines the norms under which certain actors or institutions are legitimized to have access to our private domain of the self. Seen as a predominantly (or at least historically) governmental function, identity is presented by Foucault’s biopolitics as an apparatus of control aimed at managing life, which is transformed from a private affair into a matter of policy. The state exercises its power to regulate bodies—formerly perceived as individual, distinct—as a whole, “to rationalize the problems presented to governmental practice by the phenomena characteristic of a group of living human beings constituted as a population: health, sanitation, birth rate, longevity, race” (Foucault, 1994, p.73). Cheney-Lippold describes the process of classification itself as “a demarcation of power, an organization of knowledge and life that frames the conditions of possibilities of those who are classified” (2017, p.7). This classification operates as a structure for understanding individuals and collectives from the perspective of the classifier. For this reason, identity is valuable because it legitimizes the distinction between (or within) categories based on which different privileges, freedoms, and rights are attributed.^24^ The process of transcribing analogue identity and identification in the digital, the rules of which are determined by the power relationships between the identifier and the identified, constitutes the core of the creation of digital identity (or identities).

With the growing digitalization of administration and government, digital identity became a central government concern. These efforts followed different rules and accounted for different conditions with regards identity construction, especially when compared to the ones that defined digital identity in the private sector. What eventually has become a big challenge for the state is how to negotiate the merging of different information on the citizen which have been accumulating in various different sectors of the state, without enabling unlawful discrimination and perpetuating inequality. Take for example the recent SyRi case: the Dutch Tax Agency has been using people’s nationality (Dutch/not Dutch) data as an indicator to an automated system that qualifies welfare applications as risky or not. It also processed the same nationality data of childcare benefit applicants for the purpose of combating organized fraud. The Dutch Data Protection Authority fined the Tax Agency based on personal data violations.

Ascribing digital identity systems with the responsibility to classify users, accordingly, entails the risk that important socio-political decisions become hidden behind opaque, rigid, and deterministic technological (often privately built or maintained) infrastructures. The importance and truthfulness that we attach to these categorizations makes them particularly impactful. Most importantly, these private technological infrastructures are increasingly inescapable. Control over whether one can or cannot have any digital identity is fading. It becomes clear that the entity who controls the technology controls the identity embodied in it. This process can be best understood as an ecosystem, one which is co-determined by the identity information at hand, the actors involved, the technologies and governance or design architectures which form the end digital artifact qualified as digital identity. We contend that incorporating contextual understandings of identity in the ecosystem co-creation process and ensuring the power balance between the actors involved permits transparency, accountability, and autonomy. These are both components the existence of which is necessary for a (European-wide or national) public digital identity infrastructure.

The establishment of a (digital) identity system is certainly a state responsibility, as part of its sovereign power. However, regulation of the establishment and interaction of various member state identity systems fall within the scope of responsibility of the EU. The recently adopted eIDAS Regulation^25^ creates the technical requirements and responsibility network for interoperable digital identity to function in the internal market. The element that stands out in the eIDAS Regulation is the proposal for an updated eIDAS 2.0 amendment, which facilitates cross-border electronic identification and authentication and more specifically enables the adoption of a European-wide digital identity infrastructure through the application of blockchain-based digital identity standards.^26^ In the following, we explain the blockchain-based self-sovereign ideals as implemented through European-wide policy-making.

## The Adoption of Self-Sovereign Ideals in Digital Identity Policy-Making

Digital sovereignty as expressed through claims of informational self-determination emphasizes “the autonomy of citizens in their roles as employees, consumers and users of digital technologies and services” (Pohle & Thiel, 2020). Data sovereignty is part of the strategic digital objectives of the European Union, and it is beginning to be associated with blockchain-based systems and blockchain-associated projects. As a result, blockchain is actively entering European strategic debates for reform of cross-border public service delivery and beyond. Among the solutions for which blockchain pilot projects have been launched, self-sovereign identity technological principles are introduced both in the revision of the European identity regulatory framework^27^ and as a practical architectural design for the creation of European-wide digital identity infrastructures. However, it quickly becomes apparent that the shortcomings identified in both the inscription of identity (or identities) for use by the public sector and the reuse and flow of identity (and identifying) data among different actors are not addressed by the new blockchain-based technological architecture. With citizen empowerment and self-sovereignty as its priority ideals, self-sovereign identity does not appear to be able to solve the over-capture of identifying data by public actors, or the flow of that data among different public actors. So, while the technological design appears to be susceptible to change, the new digital identity infrastructure is not promising to solve any of the historical shortcomings of identity systems created or constructed by the state. What is more, the new infrastructure is creating an environment auspicious towards the weakening of state responsibility vis-à-vis the growing datafication of its citizens.

Take, for example, the European blockchain service infrastructure (EBSI). It consists of a peer-to-peer network of interconnected nodes running a blockchain-based service infrastructure. It is the most ambitious blockchain infrastructure initiative stemming from the European Union. Launched in 2019 by the European Commission in collaboration with member states and the European Court of Auditors (united under the European blockchain partnership), EBSI is designed for cross-border government services. In the longer term, this project aims to be interoperable with other government and commercial blockchain platforms. At first glance, the EBSI represents an attempt by European institutions to engage with new technological solutions and learn how to regulate it by using it (Grech et al., 2021).

Among the first applications to be developed on the EBSI is the provision of an interoperable digital identity framework. The objective of this project is to constitute the first European blockchain infrastructure to standardize the transmission of different types of digital identifiers within the European Union. The project called European self-sovereign identity framework (eSSIF) is being developed to constitute a “generic and interoperable self-sovereign identity framework (SSI), defining the necessary specifications and building the services and supporting capacities which will allow citizens to create, control and use their own digital identity (including identification, authentication and many other types of identity-related information) without having to depend on a single centralized authority”.

The development of this project design stems from European institutions, and is based on the principles of decentralization (Bodó & Giannopoulou, 2019) and sovereignty, as institutional aspirations for the provision of services to all European citizens. However, it quickly becomes clear that these concepts are poorly defined both legally and technologically. If a distinction must be made between technological architectures promoting the centrality of the user for the provision of services and those promoting self-sovereignty, it is not obvious. Is it itself relevant? Similarly, both decentralization and people’s sovereignty are seen as the solution to combat the growing centralization of data in the hands of certain actors. However, the power relations in any infrastructure are fragile especially as citizens need the service in question to keep being provided in order to continue accessing the desired and necessary services. In addition, and most importantly, it is difficult to imagine the disempowerment of the state as a digital identity provider in favor of a self-sovereign decentralized identity which prioritizes user empowerment. As we have already established, state power (and its ensuing accountability) in identity provision can and needs to be guaranteed in any digital identity infrastructure as a trust-producing actor the value of which is derived by its legal responsibilities (Giannopoulou, 2022).

If we take the example of the pan-European infrastructure providing a blockchain service (EBSI), its success will be based on the trust that citizens have in the providers of the services and on the latter’s ability to engage the responsibility of the European institutions responsible for this infrastructure. Identifying those responsible may not always be easy, and clarification will need to be made. Decentralization, for example, does not facilitate the determination of the key players whose liability can be pursued, in particular in the event of the use of new technological architectures such as the blockchain (Finck, 2019; Giannopoulou, 2021). Moreover, as an aspiration of institutional origin at the European level, the promotion of self-sovereignty and decentralization could have the effect of disempowering the institutions responsible for developing public infrastructure and over-empowering each user/European citizen.

To illustrate this risk, the example of digital wallets can be enlightening. As their name suggests, digital wallets aim to perform the same function as their offline counterparts. They are meant to be used for storing and protecting credentials. Their function is therefore, on the one hand, to store the identifiers, to protect them against theft or prying eyes, and, on the other hand, to make them available thanks to a portable digital device, according to the needs of the holder of the this last. These wallets, which are appearing in the texts framing the implementation of digital identity, are supposed to promote the central role of their user within data transfer architectures. However, neither the documentation published by the European institutions nor the forthcoming regulations specify how the responsibilities of each actor (European Commission, member states, private actors providing the technology used, citizens) will be articulated. Without the necessary standards to regulate the provision of such digital services, users/citizens risk being left with few means of redress. This observation can be transposed when the proposed regulation is studied in the light of the rules applicable to electronic registers.

## Conclusion

Self-sovereign identity places the emphasis on individual empowerment. It recognizes the power asymmetry and the risks inherent in current digital identification infrastructures and proposes a new one focusing on, predominantly, an alternative technological design. However, the emphasis put in decentralizing the technical system of digital identity production, expression, and validation is but only one part of the process of claiming back control over our understanding of selective self-revelations vis-à-vis the state or private actors. The principles that guide self-sovereign identity, especially the user-centricity and individual empowerment run the risk of creating increased accountability of individuals in control of their information and a correlative disempowerment of other powerful actors involved in the identification processes, including public ones. This resulting imbalance can prove to be detrimental, paradoxically, to the (self-sovereign) person concerned.

The paper highlights that, overall, digital identifiers are usually not reflections of our identity. These identification processes function sometimes in concert and sometimes in tension with our identities. For this reason, digital identities can only be considered as an additional layer of identity, and not as its substitute. Thus, any effort to understand and conceptualize any digital identity while ignoring the social, economic, technological, legal, and political economy contexts which define how identities are constructed by the self and by others would be simply reductionist or superficial. This paper investigated how self-sovereign identity infrastructure provision considers (or fails to consider) the importance of technological affordances that these infrastructures might create, as well as the produced negative externalities to identity at large.

Substantially, the role of the state is versatile in identity creation, validation, authentication, and management systems. Exercising its sovereign power in (legal) identity creation, validation, and authentication, the state is also engaging in a rather complex network of identification relationships under any of the capacities: in the performance of e-government services, in the cross-border digital identification of its citizens, and in the collaboration between the public and the private sector for infrastructural support and creation of digital identity management systems. The State, guarantor of civil (digital) identity, has a formal responsibility to ensure that any digital identity infrastructure provision will not result in a disempowerment of the individual citizen and in the subsequent loss of trust in the public sector.

Finally, the paper concludes that the shifting environment of digital identity provision and management, particularly as it appears from a European, instead from a state, initiative, does not acknowledge the historical and social underpinnings that made identity creation and management necessary, and thus, it is unable to address the challenges that identity management is facing for increasingly datafied individuals.

## Data Availability

Not applicable.

## Footnotes

1 For an overview, see < https://www.theguardian.com/news/series/cambridge-analytica-files > accessed 21 October 2022.

2 See < https://theintercept.com/2021/08/17/afghanistan-taliban-military-biometrics/ > and < https://www.theguardian.com/global-development/2021/sep/07/the-taliban-are-showing-us-the-dangersof-personal-data-falling-into-the-wrong-hands > accessed 21 October 2022. For an overview of the risks related to digital state identities currently under development in emerging economies, see Renieris (2021), Why a Little-Known Blockchain-Based Identity Project in Ethiopia Should Concern Us All, Centre for International Governance innovation, < https://www.cigionline.org/articles/why-alittle-known-blockchain-based-identity-project-in-ethiopia-should-concern-us-all/ > accessed 21 October 2022.

3 Lately, numerous examples of these risks have emerged on a global scale, particularly intensified from pandemic-related public health decisions and practices. See for instance: Sato, M. (24 May 2021), Vaccine waitlist Dr. B collected data from millions. But how many did it help? < https://www.technologyreview.com/2021/05/24/1025281/covid-vaccine-waitlist-dr-b-collected-data-from-millions/ > accessed 21 October 2022.

4 The term “infrastructure” is generally used to refer to socio-technical systems that underlie or support public interest, universal or quasi-universal services (Plantin et al., 2018). Plantin et al. (2018). Infrastructure studies meet platform studies in the age of Google and Facebook. New Media & Society. 20(1):293-310. https://doi.org/10.1177/1461444816661553

5 SSI advocates frequently refer to a series of essays published by Devon Loffreto as the main elements of the ideals that would form the aspired identity system. According to The Moxy Tongue, SSI aims to decouple identity issuance by the state in order to bring it to the full control of the citizen. The Moxy Tongue (2016, February 9). Self-sovereign identity [Blog post]. *The Moxy Tongue*. < https://www.moxytongue.com/2016/02/self-sovereign-identity.html > accessed 1 November 2022.

6 Informational self-determination emphasizes the role that data protection holds in shielding individuals from interference in personal matters. The German Constitutional Court proclaimed informational self-determination, which anchored data protection in the German Constitution, a novelty of its time. This principle is “a precondition for citizens” unbiased participation in the political processes of the democratic constitutional state’Gerrit Hornung and Christoph Schnabel, “Data Protection in Germany I: The Population Census Decision and the Right to Informational Self-Determination” (2009) 25 Computer Law & Security Review 84. The European Court of Human Rights concluded that Article 8 of the European Convention on Fundamental Rights, included “the right to a form of informational self-determination, allowing individuals to rely on their right to privacy as regards data which, albeit neutral, are collected, processed and disseminated collectively and in such a form or manner that their Article 8 rights may be engaged”. ECHR, Satakunnan Markkinapörssi Oy and Satamedia Oy V. Finland, Application No. 931/13, Judgment (Merits and Just Satisfaction), Grand Chamber, European Court of Human Rights, 27 June 2017.

7 Renieris E (2020), *SSI? What we really need is full data portability.* Available online at < https://womeninidentity.org/2020/03/31/data-portability/ > accessed 1 November 2022.

8 Birch, D. (2014), Identity is the New Money, Perspectives.

9 The digital identity solutions market is predicted to be worth up to $30.5 Billion by 2024. See here < https://www.prnewswire.com/news-releases/digital-identity-solutions-market-worth-30-5-billion-by-2024--exclusive-report-by-marketsandmarkets-301004387.html > accessed 1 November 2022.

10 Sheldrake refers to Weyl’s description of “ALONE: Atomistic Liberalism and Objectivist Naive Epistemology” as a parallel to the central narrative of self-sovereign identity. “Central to ALONE is a binary between Individuals, conceptualized as largely presocial, independent ultimate loci of value / preference / good / belief (well-being for short), and some global coordination device variously referred to as the social planner, objective truth, the modeler, the mechanism designer, the impartial observer, God or, most commonly and how I will refer to it, The State”. Weyl (2020, December 15). Why I am not a Market Radical. < www.radicalxchange.org/media/blog/why-i-am-not-a-market-radical/ > accessed 1 November 2022. Cited by Sheldrake (2022), Human identity: the number one challenge in computer science, < https://generative-identity.org/human-identity-the-number-one-challenge-in-computer-science/ > accessed 1 November 2022.

11 Since the early 1990s, Donna Haraway spelled out a “cyborg” identity, to highlight that it would be increasingly difficult to discern where the individual ends and where the machine begins. Haraway (1991). The Cyborg Manifesto. In: Simians, cyborgs, and women: the reinvention of nature. Routledge. pp. 149–182.

12 As succinctly put by Bernal, “is it not my right to keep these identities separate, distinct from each other? This is not a matter of secrecy; it is a matter of choice. Arguments against such a right to compartmentalize include the idea that such compartmentalization is effectively ‘hiding’ parts of yourself— another recasting of the ‘if you’ve got nothing to hide, you’ve got nothing to fear’ argument” (2014, p.249).

13 The relationship between identification and identity becomes all the more interconnected when one considers the double conceptualization of identity as the “idem” identity (i.e., the personal aspect of the self-identity) and the ‘the ‘ipse’ identity (i.e., the social aspect to the “idem” identity). See Ricoeur (2005). The Course of Recognition. Cambridge, MA, Harvard University Press.

14 Unfortunately, identity is not defined in the official text, which -without any further explanations- uses the terminology provided by the European Commission in its communication of 19 February 2020 entitled “Shaping a digital future for Europe”.

15 Loi n° 78–17 du 6 janvier 1978 relative à l'informatique, aux fichiers et aux libertés.

16 This technological innovation would constitute, according to Bratton, the digital geography equivalent of the design choices for the postal addresses: Bratton (2015), The Stack. On software and sovereignty, MIT Press, pp 193–196.

17 See the CJEU Case C-582/14 Patrick Breyer [2016] EU:C:2016:779. The Court ruled that dynamic IP addresses are personal data, noting that “a dynamic IP address is not data related to an identified natural personal but can be considered to make log entries that relate to an identifiable person where the necessary additional data are held by the ISP”. For an overview, see Finck & Pallas (2020). They who must not be identified—distinguishing personal from non-personal data under the GDPR, International Data Privacy Law, 10(1):11–36, https://doi.org/10.1093/idpl/ipz026.

18 See < https://openid.net/ > accessed 21 October 2022.

19 See IETF (2012), The OAuth 2.0 Authorization Framework, https://datatracker.ietf.org/doc/html/rfc6749.

20 « We are increasingly going to the Web/Internet as the platform for our lives. There, our identity is not managed by the government. It’s managed, in the majority, by Facebook. When we buy things, our identity is managed by PayPal, Amazon, and Amex/Visa/Mastercard, not to mention a raft of pretenders to our identity throne, including Facebook, Google, and startups like Square. All of these are private corporations. None of them ask us for our government issued identity cards before allowing us to make a purchase. Some do ask for our SSN, of course. But online, the “government layer” is melting into the background of our identity, rather like DOS melted into the background of Windows 3. I expect this to be the source of some serious conflict in the coming decade(s).» See Battelle (2011), What Role Government, John’s Battelle’s Search Blog, 4 November 2021, Available < https://battellemedia.com/archives/2011/11/what-role-government > accessed 21 October 2022.

21 As we have noted elsewhere: There is a conflation between legal anonymity and technical anonymity. In technical terms, anonymity refers to pseudonymity together with unlinkability. In legal terms and according to Recital 26 GDPR, anonymity refers to information that cannot be related to a natural person, or that is no longer reasonably likely to be attributed to a natural person. Thus, the legal concept of anonymity, as is the case for personal data, is dynamic and context-dependent: Giannopoulou (2021), Putting data protection by design on the blockchain, European Data Protection Law Review, 7(3):388-399.

22 In his argumentation towards an establishment of a right to an identity, Bernal states that “control over the links between the online and offline identities may be the most important aspect of the rights suggested: with this in place, the ideas of when and where identities need to be verified can become clearer and more appropriate” Bernal (2011), Internet Privacy Rights, Cambridge University Press, p.237.

23 According to the 2016 United Nations (UN) E-Government survey, 148 countries provided at least one form of online transactional service, a substantial increase from previous years. United Nations (2016), United Nations E-Government Survey 2016: E-Government in Support of Sustainable Development. New York: UN.

24 The function of the “green pass” throughout the COVID-19 pandemic is a representative example of health identity data (i.e., vaccination status) being used to create different privileges among people. This example is useful to illustrate how digital identity can encompass “all of the systems and methods by which we identify ourselves through the use of digital tools in the context of specific interactions or transactions, which need not be digital—we might present an app or QR code when boarding a plane—but it is achieved at least partially through digital means” (Renieris, 2021).

25 Regulation (EU) No 910/2014 of the European Parliament and of the Council of 23 July 2014 on electronic identification and trust services for electronic transactions in the internal market and repealing Directive 1999/93/EC.

26 Proposal for a Regulation of the European Parliament and of the Council amending Regulation (EU) No 910/2014 as regards establishing a framework for a European Digital Identity.

27 Regulation (EU) No 910/2014 of the European Parliament and of the Council of 23 July 2014 on electronic identification and trust services for electronic transactions in the internal market and repealing Directive 1999/93/EC.
